# Echoes of Pink Noise: A Hypothesized Mechanism for Enhancing Sleep-Dependent Memory Consolidation with Auditory Stimulation

**DOI:** 10.1177/10738584251403967

**Published:** 2025-12-26

**Authors:** Saied Sabaghypour, Farhad Farkhondeh Tale Navi, Laura J. Batterink

**Affiliations:** 1 Department of Psychology, The University of Western Ontario, London, ON, Canada; 2 Department of Cognitive Neuroscience, Faculty of Education and Psychology, University of Tabriz, Tabriz, Iran

**Keywords:** pink noise, memory consolidation, closed-loop auditory stimulation (CLAS), hippocampal–cortical coupling, sharp-wave ripples (SW-Rs), slow oscillation (SO), spindle

## Abstract

Emerging evidence highlights the potential role of auditory stimulation in enhancing sleep-dependent memory consolidation. Pink noise appears to be an effective auditory stimulus for enhancing memory consolidation, likely due to its wide-range influence on brain oscillations. However, the specific underlying mechanisms by which pink noise enhances memory consolidation remain unclear. This perspective article presents a novel hypothesis exploring how pink noise, delivered through closed-loop auditory stimulation, may facilitate memory consolidation. Specifically, we suggest that pink noise may reach the hippocampus via the rapid auditory pathway, potentially increasing the likelihood of sharp-wave ripple (SW-R) generation. By increasing hippocampal ripple activity, the overall likelihood of synchronization with spindles and slow oscillations is also increased, enhancing hippocampal–cortical coupling. This suggests that pink noise might indirectly support slow oscillation-ripple-spindle coordination to promote systems-level consolidation and interregional information transfer. This, in turn, could enable long-term memory storage and support abstraction and generalization. Our hypothesis emphasizes a bottom-up mechanism originating from the hippocampus. Although this hypothesis currently lacks direct support from subcortical recordings, it builds on existing knowledge of sleep rhythms, hippocampal auditory pathways, and the known effects of SW-R modulation on memory formation. This perspective offers a framework for future work investigating the mechanisms by which pink noise stimulation can lead to memory enhancement.

## Sleep Rhythms and Their Role in Memory Consolidation

Sleep has long been known to provide a privileged state for memory consolidation (de Almeida-Filho et al. 2021; Geva-Sagiv et al. 2023; Rasch and Born 2013; Tilley and Empson 1978). A leading model, known as active systems consolidation, proposes that memory consolidation during sleep results from the reactivation of newly encoded memories in the hippocampus (Born and Wilhelm 2012; Diekelmann and Born 2010; Klinzing et al. 2019). The model assumes that memories are encoded in parallel in both the hippocampus and neocortex (McClelland et al. 1995). During subsequent slow-wave sleep, the repeated reactivation of memory traces in the hippocampus leads to a gradual strengthening, integration, and redistribution of these representations within neocortical networks. This information transmission between hippocampus and neocortex is thought to rely on a “triple nesting” (Klinzing et al. 2019) of key neural oscillations during slow-wave sleep—namely, slow oscillations (SOs), spindles, and hippocampal sharp-wave ripples (SW-Rs).

SOs are the most distinctive feature of the electroencephalogram (EEG) during slow-wave sleep (Steriade 2006; Steriade et al. 1993). SOs appear as high-amplitude, synchronized EEG activity at a typical frequency of ~0.8 Hz in humans (Achermann and Borbély 1997; Born and Wilhelm 2012). These oscillations are primarily generated by cortical neurons, with contributions from thalamic activity (Neske 2016; Timofeev and Chauvette 2011), and represent global neural activity alternating between depolarized, excitable up-states and hyperpolarized, quiet down-states (Massimini et al. 2003; Staresina et al. 2015). Spindles are rhythmic oscillations (12–16 Hz) produced and maintained by thalamocortical interactions (Fernandez and Lüthi 2020). Finally, SW-Rs are brief high-frequency bursts primarily observed in the hippocampus (Buzsáki 2015; Vaz et al. 2019). These bursts seem to represent field oscillations made up of hypersynchronous action potentials (Andersen et al. 1971) and typically last about 40 to 150 ms, involving widespread activation of the hippocampo-subicular network (Buzsáki 2015). Each of these oscillatory phenomena, on its own, has been functionally linked to memory consolidation. For example, SW-Rs have been associated with the reactivation of cell assemblies involved in prior encoding (Diba and Buzsáki 2007; Dupret et al. 2010), and experimental suppression of ripples has been shown to impair memory performance (Ego-Stengel and Wilson 2010). Similarly, both slow-wave sleep and spindle activity have been shown to correlate with memory consolidation (Holz et al. 2012; Mikutta et al. 2019; Schabus et al. 2004), and induction of slow waves through transcranial direct current stimulation enhanced the retention of word pairs (Marshall et al. 2011).

Importantly for the theory of active systems consolidation, each of these oscillations does not occur independently of the others but forms hierarchically nested structures (Staresina et al. 2023). Specifically, hippocampal SW-Rs convey bursts of reactivated memory traces that become temporally aligned with sleep spindles in the neocortex. The neocortex is particularly excitable during the up-state of SOs, and spindles further enhance cortical plasticity, providing windows during which hippocampal output can be effectively integrated into cortical networks (Born and Wilhelm 2012; Diekelmann and Born 2010; Helfrich et al. 2019; Jiang et al. 2019; Klinzing et al. 2019; Siapas and Wilson 1998). This nesting is thought to facilitate the communication of reactivated memory information from the hippocampus to the neocortex (Sirota et al. 2003; Staresina et al. 2015). Mounting evidence shows that not simply the presence of each signature on its own but also its synchronization is important for memory consolidation (Navarrete et al. 2020; Siapas and Wilson 1998; Staresina 2024). For example, slow-oscillation spindle coupling predicts memory consolidation (Helfrich et al. 2018; Niknazar et al. 2015), with disruption of this coupling in older adults compared to younger adults predicting impaired memory performance (Helfrich et al. 2019). In addition, memory reactivation of learned material occurs during SO–spindle complexes, and the precision of SO–spindle coupling predicts memory reactivation strength (Schreiner et al. 2021). This hierarchical nesting of oscillatory events provides a neurophysiological framework for the redistribution of memory traces (Bergmann and Staresina 2017; Schreiner and Staudigl 2020). Overall, the role of these key neural oscillations in memory consolidation is now well established, as outlined in a number of comprehensive reviews (Klinzing et al. 2019; Marshall et al. 2020; Staresina 2024).

## Benefits of Pink Noise in Sleep-Dependent Memory Consolidation

The functional significance of SOs in memory consolidation has led to efforts using external auditory stimulation, especially using *pink noise*, to enhance such activity (Harrington and Cairney 2021; Ngo et al. 2013; Ngo et al. 2015). Pink noise (see Box 1) is a type of sound characterized by its spectral density (*S*(*f*)) being inversely proportional to frequency (1/*f*) (Yang et al. 2015). Unlike white noise, in which power is equally distributed across frequencies, pink noise resembles sounds commonly found in biological systems and in natural environments, such as steady rain (Szendro et al. 2001; Yang et al. 2015). Interestingly, compared to quiet conditions, the presentation of continuous pink noise reduced brainwave complexity during wakefulness, as measured by EEG (Zhou et al. 2012). It also improved the percentage of stable sleep and overall sleep quality, as measured by electrocardiography and subjective ratings (Zhou et al. 2012). It is well established that sleep quality is linked to memory consolidation, with poor, disrupted, or fragmented sleep negatively affecting the consolidation of memories (e.g., Djonlagic et al. 2012; Hokett et al. 2021; Prince and Abel 2013; Tempesta et al. 2015). Thus, one route by which pink noise may benefit memory consolidation processes is simply by improving sleep quality. A systematic literature review on the effects of auditory stimulation on sleep outcomes further supports the specific advantage of continuous pink noise presentation for improving sleep, showing improved sleep outcomes in 82% of studies, compared to 33% for white noise (Capezuti et al. 2022).

**Box 1.** What Is Pink Noise?.

The name “pink noise” comes from the fact that visible light with a similar 1/*f* power distribution appears pink in color. Pink noise is a type of sound characterized by a frequency spectrum where power decreases as frequency increases. This means that lower frequencies carry more energy than higher ones. Unlike white noise, which has equal power across all frequencies and can sound harsh (like TV static), pink noise resembles natural ambient sounds such as steady rain or ocean waves. 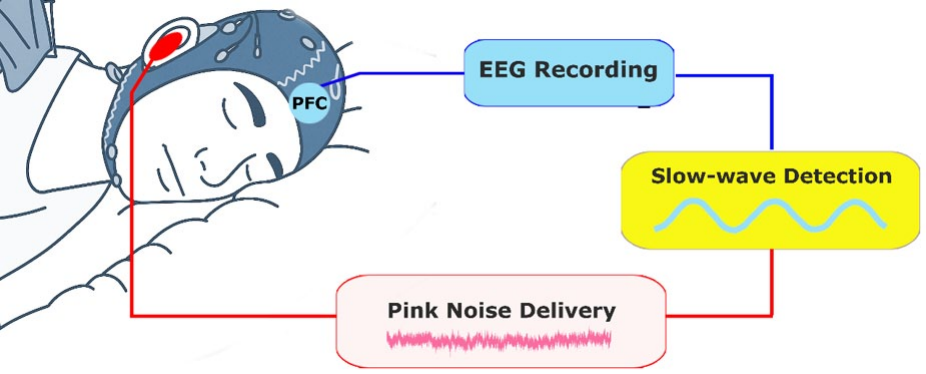 In closed-loop auditory stimulation paradigms, pink noise can be delivered precisely at the upstate of SOs and thereby enhance amplitude of SOs, which are important for memory consolidation.

While continuous pink noise may improve overall sleep quality in a general manner (Zhou et al. 2012), *discrete pulses* of pink noise delivered at target brain states—particularly the up-state of SOs in nonrapid eye movement (NREM) sleep—have been shown to enhance distinct sleep oscillations, including SOs (~0.5–1 Hz), thereby improving memory consolidation (Esfahani et al. 2023; Leminen et al. 2017; Ngo et al. 2013; Ngo et al. 2015; Papalambros et al. 2017). Using *closed-loop auditory stimulation* (CLAS), brain activity measured with EEG is continuously monitored, and the timing of auditory stimuli is dynamically adjusted to coincide with target brain states (Jaramillo et al. 2024). Pioneering CLAS studies by Ngo and colleagues (2013, 2015) showed that pulses of pink noise—timed to coincide with the excitable up-phase of ongoing SOs—enhanced such rhythms during NREM sleep and improved declarative memory consolidation, as measured by a word-pair learning task. A number of subsequent studies have confirmed these findings, reporting that pink noise relative to sham stimulation enhances slow-wave activity (Ngo et al. 2013; Papalambros et al. 2017; Salfi et al. 2020; Simor et al. 2018; Zeller et al. 2024), spindle activity (Choi et al. 2018; Choi and Jun 2022), and memory consolidation (Harrington and Cairney 2021; Leminen et al. 2017; Papalambros et al. 2017; Wunderlin et al. 2024), though improved memory consolidation is not always observed (Henin et al. 2019). Moreover, recent evidence suggests that the efficacy of CLAS can vary with age, with older adults showing reduced susceptibility and smaller electrophysiological responses compared to younger participants (Schneider et al. 2020). In head-to-head comparisons, pink noise has been shown to evoke larger-amplitude SOs, broader spatial activation, and significantly lower habituation over repeated stimulations compared to other types of auditory stimuli such as narrower-spectrum pure tones and spoken vowels, providing evidence of a specific advantage of pink noise for enhancing sleep oscillations (Debellemanière et al. 2022).

Alongside the CLAS studies showing memory benefits of pink noise presentation, a largely separate line of research has developed in parallel, in which auditory cues linked to a wake learning episode are subsequently presented during sleep (e.g., Antony et al. 2012; Rudoy et al. 2009; for meta-analysis, see Hu et al. 2020). This approach, known as targeted memory reactivation (TMR), enhances memory performance across numerous domains (Carbone and Diekelmann 2024). To facilitate learning, TMR typically incorporates semantically meaningful cues that can be easily linked to learned information (e.g., naturalistic sounds such as a cat meow or kettle whistle; Rudoy et al. 2009). Closed-loop TMR has also been applied, in which learned cues are delivered during the up-phase of SOs (Göldi et al. 2019; Ngo and Staresina 2022; Nicolas et al. 2025). Similar to pink noise pulses, TMR cues delivered during the SO up-state have been shown to enhance SO amplitude, spindle activity, and SO–spindle coupling (Göldi et al. 2019; Ngo and Staresina 2022; Nicolas et al. 2025) and to improve memory retention (Ngo and Staresina 2022). However, it is important to note that a number of TMR studies have reported null effects on memory performance or even unintended outcomes, such as increased forgetting or selective prioritization of certain memories over others, indicating that the efficacy of TMR may depend on factors such as memory strength, cue specificity, and sleep stage (Cairney et al. 2016; Carbone et al. 2023; Hu et al. 2020; Wick and Rasch 2023). Overall, while both pink noise and auditory TMR cues influence neural dynamics, the underlying mechanisms by which they do so are presumably distinct: TMR targets content-specific reactivation and depends on prior learning, whereas pink noise modulates the broader physiological state and is independent of prior learning. Notably, when TMR cues and pink noise are matched for perceived volume, TMR cues have relatively lower energy in their spectral content, which is relevant given prior work showing that pink noise elicits larger SOs than more narrowband sounds (Debellemanière et al. 2022). However, whether pink noise might confer specific advantages over meaningful, content-specific TMR stimuli remains to be tested in well-controlled experimental comparisons.

The exact mechanisms by which acoustic stimulation enhances SOs are not yet understood. One hypothesis is that phase-locked auditory input may synchronize larger populations of neurons with the ongoing SO rhythm (Navarrete et al. 2020). Specifically, well-timed auditory stimulation may trigger near-synchronous depolarization of widespread cortical neurons, which would then be followed by a massive hyperpolarization, leading to slow waves with larger amplitudes that recruit a more widespread network compared to spontaneous slow waves (Bellesi et al. 2014). According to the active system consolidation model, the depolarization up-phase of the SO then drives spindles and hippocampal SW-Rs, creating spindle-ripple events that support effective information transfer between the hippocampus and cortex (Born and Wilhelm 2011; Molle and Born 2011). This idea is supported by human intracranial data showing SO up-states establish a coarse time window for spindles and ripples to coincide and that spindle occurrence raises firing rates to a threshold that triggers ripples (Staresina et al. 2023). However, SOs are not the only factor that can trigger ripples. In addition to this top-down mechanism—wherein auditory-enhanced SOs temporally organize spindles and ripples—there may also be parallel bottom-up mechanisms, in which auditory stimulation directly engages the hippocampus via subcortical routes. As will be discussed further below, we propose that auditory stimulation during SO up-states could directly increase the likelihood of ripple generation through the entrainment of hippocampal activity. In the remaining part of the article, we present a brief review of how auditory stimulation might affect hippocampal processes through the involvement of the rapid auditory pathway, before presenting our novel hypothesis in more depth. Finally, we conclude by discussing broader implications and potential recommendations for future studies.

### Effects of Auditory Stimulation on the Hippocampus

**Figure 1.**
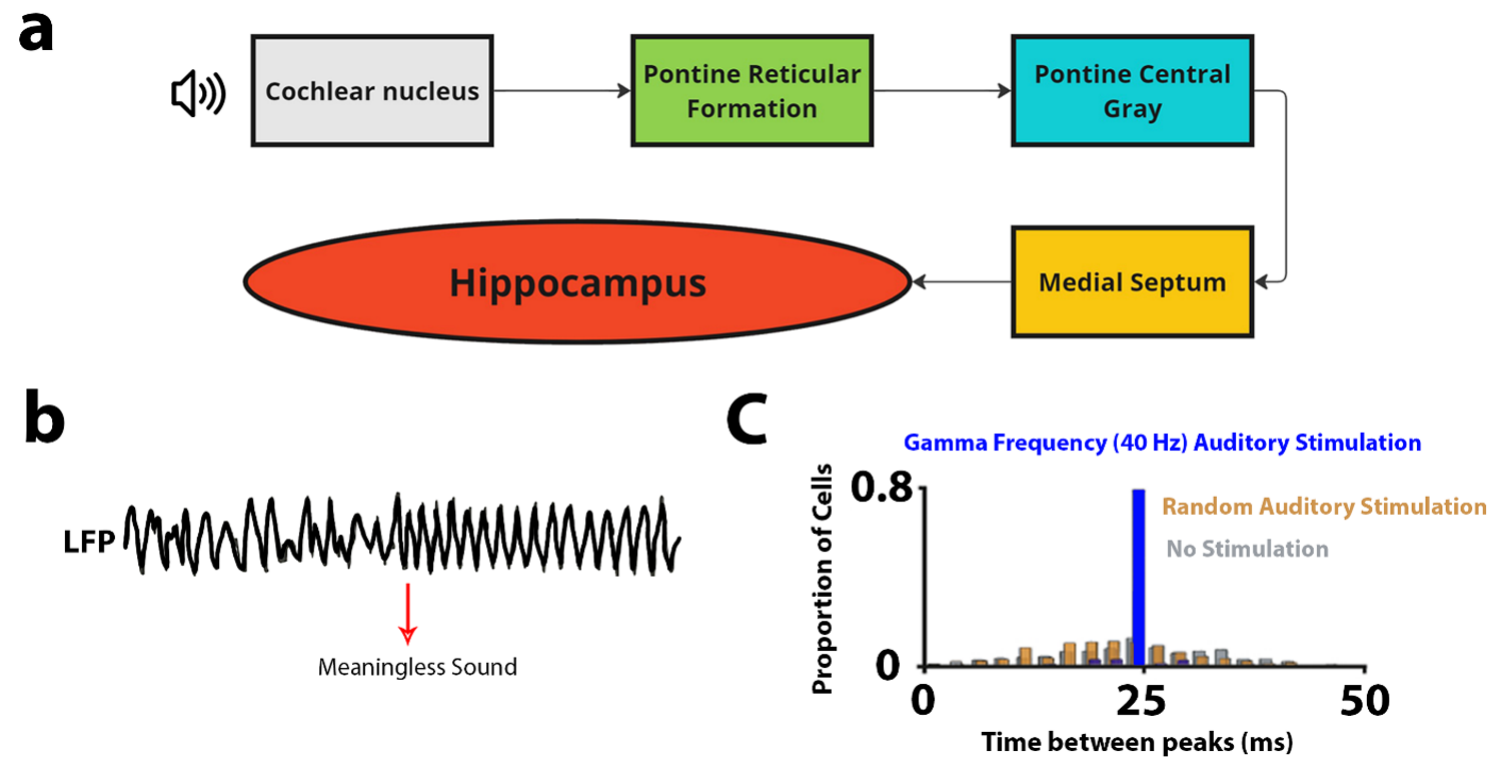
The rapid auditory pathway and demonstrations of auditory stimulation effects on the hippocampus. (a) The rapid auditory pathway containing 5 synapses. (b) Hippocampal Local Field Potential (LFP) reactions in cats in response to meaningless auditory stimulation (whistle or hiss) (adapted from Green and Arduini 1954). (c) Peak firing rates in CA1 hippocampal cells in rats during 40-Hz gamma band stimulation (corresponding to a 25-ms period; blue), random stimulation (orange), and no stimulation (gray) (adapted from Martorell et al. 2019).

The hippocampus receives processed input from all sensory modalities, including audition (Aly and Turk-Browne 2018). It is interconnected through the well-known trisynaptic loop, which routes signals from the entorhinal cortex (ERC) through the dentate gyrus (DG), CA3, and CA1, before returning to the ERC and then the neocortex (Billig et al. 2022; Guzman et al. 2021). Additionally, there are direct projections from the ERC to CA1 (monosynaptic pathway) and recurrent connections within CA3 (Basu and Siegelbaum 2015). The hippocampus also connects reciprocally via the fornix to the thalamus, mammillary bodies, basal forebrain, amygdala, basal ganglia, and various cortical regions, including the cingulate, frontal, and parietal lobes (Insausti and Amaral 2004; Irle and Markowitsch 1982). Auditory input can be integrated with other sensory modalities at various stages throughout this synaptic network. Bypassing the primary auditory cortex, subcortical pathways transmit auditory information directly to the hippocampal formation, including a route from the cochlear nucleus through the pontine nuclei and medial septum (Xiao et al. 2018), known as the *rapid auditory pathway*. These subcortical pathways likely offer rapid, broad communication of sound presence (Fig. 1a), in contrast to slower cortical routes that deliver more detailed and integrated representations of sound and its meaning (Rolls 1996).

In addition to anatomic evidence, there is also functional evidence that the hippocampus is responsive to auditory stimuli, as reflected by hippocampal neural responses triggered by auditory stimuli encountered under passive listening conditions (see Fig. 1b). For instance, in cats, increased theta has been observed in response to meaningless stimuli such as whistles (Green and Arduini 1954). In humans, pure-tone presentations reset the phase of ongoing hippocampal oscillations (Martorell et al. 2019; Rosburg et al. 2007), and auditory tone stimulation presented in the gamma range drives neural activity in the auditory cortex and hippocampal CA1 (see Fig. 1c) (Martorell et al. 2019). A change in auditory stimulus intensity as short as 1 ms results in changes in spike correlations between the hippocampal region and the auditory cortex (Sakurai 1996), highlighting the very sensitive nature of the hippocampus to auditory stimuli. Furthermore, the level of synchrony among groups of neurons in the hippocampal CA1 region varies across different auditory stimulation frequencies. Sub-millisecond synchrony occurs most prominently at preferred frequency ranges, including 140 to 200 Hz. This suggests that the hippocampus may be selectively responsive to specific frequency bands (Takahashi and Sakurai 2009). This synchrony among hippocampal neurons provides a foundation for exploring its role in enhancing memory consolidation mechanisms.

## Pink Noise in Memory Consolidation: A Hypothesized Mechanism

**Figure 2.**
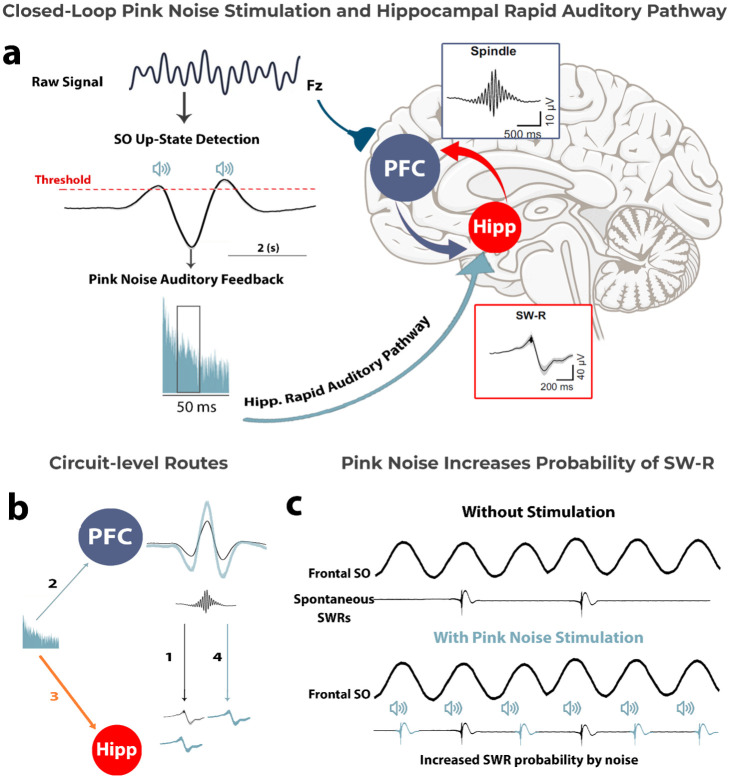
Proposed hypothesis: Pink noise enhances memory consolidation by increasing SW-R probability and thus SO–SW-R coupling. (a) Closed-loop auditory stimulation with pink noise enhances memory consolidation via hippocampal–cortical coupling. SOs are detected during their up-state, and pink noise auditory feedback is delivered. The pink noise is processed through the hippocampal rapid auditory pathway, increasing the likelihood of SW-R generation in the hippocampus, a critical process for memory consolidation. SW-Rs synchronize with spindles, facilitating hippocampal–cortex communication. (b) A circuit-level representation of the proposed pink noise stimulation effect. Path number 1 indicates spontaneous SW-R activity in the hippocampus, which may be independently influenced by SO amplitude in the PFC. Pink noise is known to increase SO amplitude in the PFC (path number 2; e.g., Ngo et al. 2013) and—as proposed in this article—is hypothesized to facilitate SW-R generation in the hippocampus via the rapid auditory pathway (path number 3). Indirectly, there is also a possibility that increasing SO amplitude along with spindle activity might enhance SW-R generation (path number 4). (c) Illustration of the proposed effect of pink noise stimulation on increasing the probability of SW-R generation in the hippocampus. Hipp, hippocampus; PFC, prefrontal cortex; SO, slow oscillation; SW-R, sharp-wave ripple.

Crucially, as reviewed above, pink noise has emerged as a form of stimulation for memory consolidation in CLAS studies (Debellemanière et al. 2022; Ngo et al. 2013), without requiring prior cue–memory associations. Nonetheless, the specific mechanism(s) by which pink noise enhances memory consolidation remain an open question. Building on the growing body of evidence reviewed above, we propose a novel hypothesis to account for how pink noise facilitates memory consolidation. Specifically, building on the assumption that pink noise reaches the hippocampus via the rapid auditory pathway (Billig et al. 2022) (third path; Fig. 2b), we hypothesize that pink noise could increase the likelihood of SW-R generation by directly entraining hippocampal activity. In turn, this may increase the overall likelihood of a synchronized SO–spindle–SW-R event, enhancing hippocampal–cortical coupling and supporting effective memory consolidation.

**Figure 3.**
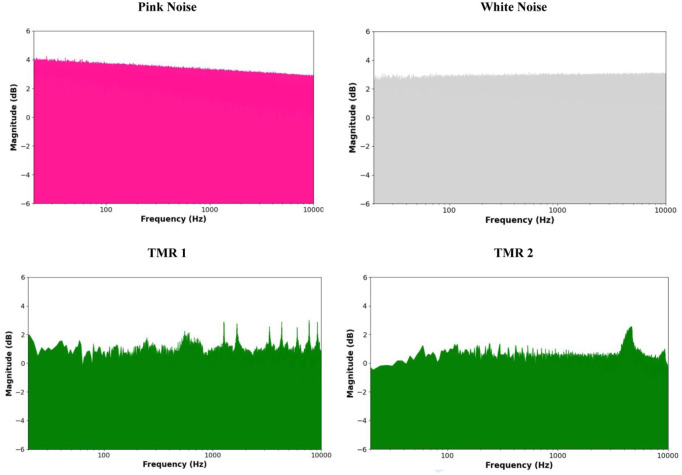
Spectral comparison of normalized pink noise, white noise, and 2 targeted memory reactivation (TMR) stimuli (left: bell ring, right: bird chirp), showing log-scaled magnitudes derived from FFT after peak normalization. Magnitudes were computed as the absolute value of the Fast Fourier Transform (FFT) and log-transformed to visualize the spectral content (TMR files adapted from Oudiette et al. 2013).

SOs provide a time window of opportunity for SW-Rs to occur, but they do not always spontaneously appear during these windows (Henin et al. 2019; Staresina et al. 2015). According to our proposed hypothesis, pink noise may enhance the likelihood that SW-Rs will co-occur within these optimal windows by directly inducing SW-Rs. In particular, the rapid auditory pathway (see Fig. 1a) may facilitate the delivery of pink noise’s relevant frequencies to the hippocampus. This auditory input could help promote the generation of SW-Rs, increasing overall coupling between the hippocampus and cortex. The enhanced synchronization would, in turn, support the consolidation of memories by aligning hippocampal and neocortical oscillatory activity during sleep (see Fig. 2a), as reviewed earlier (see also Klinzing et al. 2019; Mikutta et al. 2019; Staresina et al. 2015). Relative to other auditory stimuli, pink noise may be particularly effective at targeting hippocampus-relevant frequencies due to its broad frequency spectrum and its characteristic higher energy concentration at lower frequencies (see Fig. 3). The hippocampal SW-Rs that are crucial for memory consolidation fall within the 100- to 200-Hz range in primates and humans (Bragin et al. 1999; Staresina et al. 2015)—an area where pink noise has a relatively higher energy compared to other sounds. This overlap suggests that pink noise may effectively stimulate hippocampal ripple activity through direct entrainment at the relevant frequencies. This proposal also raises questions about how precisely timed stimulation protocols might optimize these processes, a topic we briefly explore further in Box 2.

**Box 2.** Sleep Consolidation Quantum.

The exploration of sleep-dependent memory consolidation reveals parallels with theta rhythm dynamics (Hyman et al. 2003). Early CLAS studies showed that presenting more than 2 auditory clicks provided no added benefit, in terms of driving SOs as neural responses entered a refractory period (Ngo et al. 2015). Although that study focused on enhancing slow oscillations and reducing seizure risk, it also suggested that excessive stimulation might limit neural responsiveness. This observation aligns with the idea of a “sleep consolidation quantum,” inspired by findings from hippocampal theta activity and memory consolidation. The theta rhythm is critical for synaptic plasticity, with long-term potentiation (LTP) maximized at the peak of the theta cycle and minimized at its trough (Huerta and Lisman 1993; Hyman et al. 2003). Brief, rhythmic bursts of stimulation—rather than sustained input—can suffice for effective memory encoding, as shown by studies reporting that 2 high-frequency bursts spaced ~200 ms apart can induce long-term synaptic changes (Kropotov 2010).

A schematic view of the known and our proposed hypothetical effects of pink noise on the cortex and hippocampus is presented in Figure 2b. Since SW-Rs are closely tied to memory consolidation, the hypothesis that pink noise could promote their occurrence—and thus enhance hippocampal–cortical coupling—aligns with the neural processes reviewed in this article. As can be seen in Figure 2b, we outline several plausible routes: path 1 depicts spontaneous SW-Rs in the hippocampus. Pink noise can directly enhance SO amplitude in the prefrontal cortex (path 2), which in turn may promote spindle generation through thalamocortical loops and possibly facilitate hippocampal SW-Rs (path 4). This aligns with prior work showing that SOs temporally organize spindles (Latchoumane et al. 2017; Ngo et al. 2013). Therefore, while we hypothesize that auditory stimulation may initiate ripple activity via subcortical routes, at the same time, enhanced SO and spindle activity—modulated by the thalamus—may feed back to influence ripple timing (path 4). In parallel, path 3 represents a bottom-up route: pink noise stimulation may reach the hippocampus via the rapid auditory pathway. This rapid auditory pathway—from the cochlear nucleus through the pontine nuclei and medial septum to the hippocampus—contains 5 synapses (see Fig. 1a). Based on transmission times observed in key node structures of this pathway, including the brainstem reticular formation, medial septal nucleus, and hippocampal CA3 region (Krause et al. 2003; Moxon et al. 1999), hippocampal responses would be expected to emerge within 5 to 15 ms of sound onset. This direct subcortical route (i.e., rapid auditory pathway), with its short latency and small number of synapses, makes ripple-range entrainment plausible by ensuring rapid and synchronized input to the hippocampal formation (Manis 2008). In contrast, cortical pathways may influence ripple likelihood more indirectly, by strengthening SO–spindle coupling and thereby modulating the timing of hippocampal reactivation (Sanda et al. 2021). Overall, our hypothetical mechanism may offer a logical next testing target for research into the facilitation of memory consolidation through acoustic stimulation. In particular, whether such auditory stimulation, compared to other noises, induces greater interaction in neurons of the hippocampus and neocortex during sleep remains to be explored.

## Summary and Future Directions

In this article, we outline a novel hypothetical mechanism through which pink noise during CLAS may enhance memory consolidation. The synchronization of SOs and hippocampal SW-Rs is crucial for memory processes, as the coupling between hippocampal and cortical regions appears to drive the consolidation of newly acquired information. Again, although SOs alone may not be sufficient for memory consolidation, the co-occurrence of SOs, SW-Rs, and sleep spindles in the hippocampus and cortex creates the neural framework necessary for this process.

Here, we hypothesized that pink noise may facilitate this synchronization. By potentially engaging the hippocampus via rapid auditory pathways, pink noise may help facilitate or modulate SW-R activity, which in turn synchronizes with spindles generated in the cortex, thereby enhancing hippocampal–cortical coupling. The engagement of a broader range of cortical and subcortical networks, as observed in previous studies, suggests that pink noise could enhance neural synchronization, potentially improving the consolidation of memory during sleep. However, it is important to note that this hypothesis remains speculative. No studies to our knowledge have recorded subcortical activity directly in response to pink noise during sleep to confirm these processes. Future studies (see also Box 3) could utilize intracranial EEG (iEEG) in patients with epilepsy (Mercier et al. 2022; Parvizi and Kastner 2018), employing depth electrodes to capture both field potential and neuronal firing during natural sleep. Recording unit and field activity from multiple sites in the hippocampus and neocortex could provide insights into the temporal dynamics of pink noise–induced neural synchronization. If our bottom-up hypothesis is correct, we would expect iEEG recordings to show pink noise evoking hippocampal SW-Rs with latencies preceding cortical spindle activity that would suggest a hippocampal-driven cascade. Importantly, such recordings could also reveal causal interactions by determining whether pink noise directly triggers SW-R generation in the hippocampus via rapid auditory pathways and assessing whether these events influence SOs and spindle activity in the neocortex.

**Box 3.** Critical Predictions and Experiments.

Here, several testable predictions and experimental approaches—primarily using intracranial (iEEG) recordings—are outlined to empirically evaluate the proposed hypothesis: **Pink noise–evoked SW-R occurrences:** SW-Rs preferentially occur during the up-state phase of SOs with longer durations and are suppressed during down-states (Mölle et al. 2006; Yamada et al. 2025). We therefore predict that brief pink noise pulses (single clicks, ~50–100 ms) delivered during SO up-states will increase SW-R occurrences. I-EEG recordings from the hippocampus during NREM sleep could be combined with CLAS to directly test this prediction. **Spectrum-controlled contrasts:** To determine whether pink noise confers an advantage in modulating hippocampal SW-R activity, carefully controlled stimulation contrasts could be designed by constructing pink, white, and band-limited auditory stimuli—in addition to environmental sounds typically used in TMR studies—with matched sound pressure levels (SPL). This approach would isolate the specific role of spectral composition and clarify whether the spectral characteristics of pink noise confer a distinct advantage for modulating hippocampal SW-R activity compared with other sounds. Consequently, if pink noise influences hippocampal activity through its spectral richness, one would expect generalized increases in ripple probability or SO–spindle–ripple synchronization, independent of prior learning. In contrast, TMR cues should elicit ripple–spindle coupling that selectively reactivates task-relevant neural ensembles. **Directionality (lead-lag tests) of hippocampal–cortical coupling:** If pink noise primarily drives hippocampal activity (e.g., as in path 3; Fig. 2), SW-R events should precede cortical spindles in a bottom-up fashion. Conversely, a top-down process (e.g., paths 2 and 4) would be indicated if slow oscillations or spindles occur before the hippocampal SW-R response. This prediction can be tested by computing cross-correlations or directed connectivity (e.g., Granger causality or transfer entropy) around pink noise pulses to compare the temporal direction of ripple→spindle versus spindle→ripple activity. **Refractory dynamics with double-pulse CLAS:** We predict that closely spaced double pulses (e.g., 0- to 250-ms interstimulus intervals) will produce a refractory pattern, where the second pulse might elicit a reduced ripple response. This approach is in line with previous findings that CLAS exhibits self-limiting dynamics (Ngo et al. 2015), which might also extend to hippocampal ripple responses with comparable refractory properties in subcortical circuits. To examine potential refractory effects in hippocampal SW-R generation, stimulation experiments could use single and double pink noise pulses with systematically varied interstimulus intervals.
